# Methods for detecting and monitoring Salmonella infection and chronic carriage in living mice using bioluminescent in vivo imaging

**DOI:** 10.1099/acmi.0.000913.v3

**Published:** 2024-11-29

**Authors:** Aliyah N. Bennett, Baileigh Laipply, John S. Gunn

**Affiliations:** 1 Center for Microbial Pathogenesis, Abigail Wexner Research Institute at Nationwide Children’s Hospital, Columbus, OH, USA; 2 Infectious Diseases Institute, The Ohio State University, Columbus, OH, USA; 3 Department of Pediatrics, College of Medicine, The Ohio State University, Columbus, OH, USA

**Keywords:** *Salmonella*, biofilm, bioluminescent, chronic carriage, *in vivo* imaging

## Abstract

*Salmonella enterica* serovar Typhi primarily persists in chronic carriers by forming biofilms on gallstones in the gallbladder. We have developed a gallstone mouse model to study chronic carriage. To better understand the infection timeline and differentiate between mice that have maintained long-term gallbladder carriage from those that have cleared infection, we utilized bioluminescent *S*. Typhimurium and *in vivo* imaging to detect and track the organ-specific presence of bacteria in living mice. The mice infected with our bioluminescent *S*. Typhimurium showed luminescence in the abdomen as early as 3 days in comparison to the mice infected with non-luminescent WT *S*. Typhimurium. With our methods, we achieve image resolution such that we can confidently identify the presence of *S*. Typhimurium in the gallbladder at >60 days post-infection. Using these methods, we have determined that the minimum number of bacteria necessary to detect luminescence in the mice is 10^3^ c.f.u. and that one out of six initially infected mice will remain persistently infected for greater than 60 days, with gallbladder bacterial loads reaching upwards of 10^3^ per milligram of tissue. Given that our limit of detection of luminescence is 10^3^ c.f.u., our sensitivity is robust enough to identify the bacterial loads present in the average chronically infected mouse. The quantification of individual organs’ bacterial c.f.u. and comparison of luminescence between WT and luminescent *S*. Typhimurium validate that our technique is specific and sensitive enough to detect organ-specific infection in our model of typhoidal chronic carriage.

## Data Summary

One supplementary figure and all data used to generate figures for this article can be accessed at FigShare https://doi.org/10.6084/m9.figshare.26937931.v3.[1].

## Introduction

Bioluminescent and fluorescent *in vivo* imaging systems are a powerful tool for non-lethally detecting and monitoring infection in animal models. Many clinically relevant animal models of infection have utilized bioluminescent imaging in order to track infection and monitor the efficacy of treatment [2,7]. *In vivo* imaging tools have also been used to identify unexpected sites of infection in living animals that would have, otherwise, required euthanasia and necropsy to identify, which also limits the ability to track progression of disease [2,8, 9].

We have developed a murine model of typhoidal chronic carriage by infecting NRAMP1^+/+^(*SLC11A1*) mice (129X1/SvJ) with gallstones that are induced by a 6–8-week lithogenic diet with *Salmonella* Typhimurium [10,12]. Following infection, the bacteria are phagocytosed by macrophages, but not successfully killed, and are consequently transported to the liver and traverse into the gallbladder and intestines to model the natural course of chronic infection and gallbladder carriage [13,14]. This murine model recapitulates the human chronic typhoidal infection state, specifically of *Salmonella* biofilm production on gallstones, and has allowed us to test anti-biofilm therapies that may be used to treat the chronic carrier state [15,16]. Notably, this chronic infection is asymptomatic in mice as well as in humans and difficult to detect without sacrificing the mouse and measuring bacterial c.f.u.

**Fig. 1.**
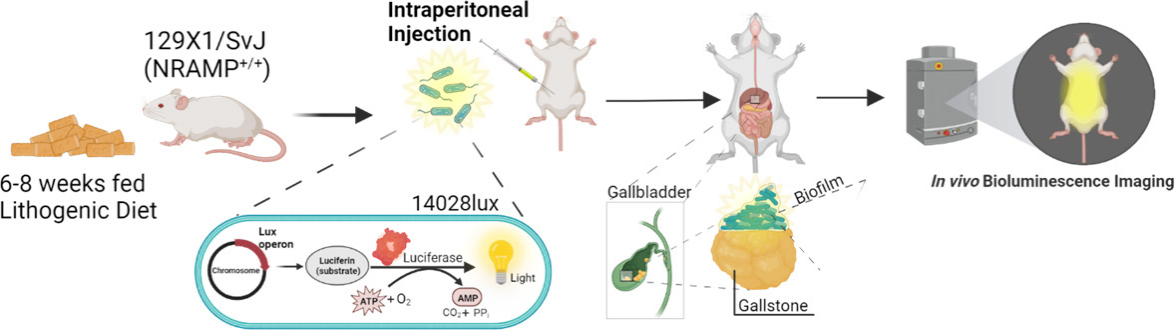
Infection and *in vivo* imaging of mice with bioluminescent *Salmonella*. Created with Biorender.com.

Concerns have been raised that bacteria in oxygen-restricted environments, such as the gastrointestinal (GI) tract or abscesses [2,9, 17], or metabolically limited bacteria in a biofilm, may be limited in their ability to luminesce at detectable levels [17]. Other groups have demonstrated the ability to observe bioluminescence in the GI tract [2,18], specifically in the gallbladder [9,19]. However, to date, no one has observed bioluminescent *Salmonella* in the murine gallbladder, and given the dual challenge of a low-oxygen environment and the biofilm state of typhoidal gallbladder infection, we aimed to develop a method for detecting and monitoring chronic gallbladder infection in our murine model of chronic typhoid fever. Here, we describe a method for establishing and monitoring chronic *Salmonella* infection in the gallbladder and other organs (Fig. 1).

## Methods

### Bacterial strains and phage transduction

Bacterial strains used were *S.* Typhimurium 14,028 and *S*. Typhimurium Xen33 (Perkin Elmer, Waltham, USA). P22 transduction was employed to introduce a chromosomal insertion of the Xen33 *lux* operon into *S*. Typhimurium 14,028 showing constitutive expression of bioluminescence. Luria–Bertani (LB) broth with 40 µg ml^−1^ kanamycin (LB+Kan) was inoculated with a single colony of *S*. Typhimurium Xen33, the donor strain, and incubated at 37 °C in a rolling drum with aeration overnight. After incubation, fresh p22 was diluted 1:2000 into the culture and re-incubated overnight to allow for phage replication. Following incubation, the remaining cells and cellular debris were removed with chloroform. The phage was introduced to the *S*. Typhimurium 14,028 (WT), the recipient strain, and incubated for 25 min at 37 °C to allow for a single round of infection and recombination event. The phage was then adsorbed with EGTA in addition to selecting the desired *lux* insertion by plating the bacteria on LB+Kan agar and incubating at 37 °C overnight. The resulting colonies were serially streaked over 3 days on LB+Kan agar with EGTA in order to fully adsorb phage. In order to exclude any pseudo-lysogenic cells that may still be present, candidate colonies were streaked perpendicular to lytic P22 phage on Evans Blue-Uranine agar. Uninfected cells will lyse after crossing the P22 line, resulting in decreased growth and pH that presents as darker-coloured colonies [20]. The resulting isolate was confirmed to have the full *lux* operon by incubating it in LB+Kan broth and measuring luminescence in a SpectraMax M3. This isolate is referred to as ‘14,028 lux’ and stored in 20% glycerol at −80 °C for future use.

In order to identify the location of the *lux* operon insertion in 14,028 lux and to identify any unexpected mutations as a result of the transduction, both WT and 14,028 lux were subjected to whole-genome sequencing and compared for variants. Sequencing and assembly of the WT and 14,028 lux isolates was performed by SeqCoast Genomics (Portsmouth, NH, USA). In brief, samples were extracted using the MagMAX Microbiome Ultra Nucleic Acid Isolation Kit. Sequencing was performed both on the Illumina NextSeq2000 platform using a 300 cycle flow cell kit to produce 2×150 bp paired reads and the GridION platform using a FLOW-MIN114 Spot-ON Flow Cell, R10 version with a translocation speed of 400 bp. Open source software [Trimmomatic (version 0.39), Unicycler (version 0.4.4) and Porechop (version 0.2.4)] were used to assemble the genomes [21,25]. Following assembly, the genomes were aligned, and the variants were identified using the Mauve algorithm in MegAlign Pro with NC_016856.1 as a reference for features. The kanamycin resistance cassette present in the *lux* contruct was mapped to the 14,028 lux sequence using SeqBuilder Pro.

### Mouse infection

#### Murine model of typhoidal infection

A total of 40 adult 129X1/SvJ mice (The Jackson Laboratory) were used in this study. Mice were fed a lithogenic diet (conventional mouse chow supplemented with 1% cholesterol and 0.5% cholic acid, Envigo) for 6–8 weeks in order to induce gallstone formation. After the initial diet period, mice were maintained on a diet of conventional mouse chow for the remainder of the experiment. To prepare the infection inoculum, overnight cultures of WT and 14,028 lux *S*. Typhimurium were grown in LB and LB+Kan liquid media, respectively, prior to being normalized and diluted in sterile PBS. The mice were injected with 200 µl (1.1×10^4^–1.6×10^4^ c.f.u.) of either WT or 14,028 lux into the peritoneum or an equal volume of sterile PBS. Previous studies support that intraperitoneal (i.p.) infection provides a reliable and reproducible systemic infection that results in asymptomatic infection as well as gallbladder carriage [10,26, 27]. The inoculum was confirmed by serially diluting and then drip plating each inoculum to LB or LB+Kan agar and incubating overnight at 37 °C, followed by enumeration of the resulting colonies.

### Bacterial competition

Gallstones were induced in nine female mice as described above in two independent experiments. Animal numbers were determined based on alpha cut-off of 0.05, requiring *n*=8 to be sufficiently powered to detect any differences in fitness. The bacterial inoculum was created by diluting separate overnight cultures of 14,028 lux and WT *S*. Typhimurium in sterile PBS to ≈2.5×10^3^ c.f.u. ml^−1^ and then combining equal volumes of each diluted culture to create a final inoculum of 2.5×10^4^ c.f.u. ml^−1^ of total bacteria. Each mouse was given an i.p. injection with 200 µl of the inoculum for a total infectious dose of 5×10^3^ c.f.u. Following infection, the c.f.u. of each strain were enumerated and determined to be 2.5×10^3^–3.90×10^3^ c.f.u for 14,028 lux and 2.4×10^3^–20.6×10^3^ c.f.u. for WT. Seven days post-infection, mice were euthanized via CO_2_ inhalation, and the gallbladder, liver, spleen, caecum, kidney and whole blood were aseptically collected for c.f.u. enumeration. In brief, each organ was homogenized in sterile PBS using bead beating and then serially diluted and drip plated on both Xylose Lysine Deoxycholate (XLD) and XLD with 40 µg ml^−1^ kanamycin (XLD+Kan) agar and incubated overnight at 37 °C. *Salmonella* form black colonies on XLD agar, allowing for sensitive identification of *Salmonella* against other bacterial species that may also be present in non-sterile organs. All black colonies were counted on each plate, and the sum of c.f.u. on the XLD+Kan agar was subtracted from the c.f.u. on the XLD agar without Kan to determine the proportion of each strain present in each organ. The c.f.u. were normalized to the mass of tissue, and then, the competition index for each organ was calculated to identify any differences in fitness between the WT and 14,028 lux strains. Statistical analysis was performed with GraphPad Prism 9.

### Mouse anaesthesia and imaging

Mice are imaged using the Xenogen Spectrum CT. Prior to imaging, mice were anaesthetized in an induction chamber with 1% isoflurane. Following sedation, mice were transferred to the imaging chamber, and anaesthesia was maintained using isoflurane supplied through nose cones for the duration of imaging. After imaging was complete, mice were returned to their cages and monitored for recovery from anaesthesia. Using Living Image software, photographic and bioluminescent images were acquired on ‘auto’, which adjusts exposure time, pixel binning and f-stop for optimal image quality and prevents signal oversaturation. To limit excess anaesthesia exposure, the exposure time was limited to a range of 0.5 s to 5 min. For 2D imaging, black paper and black barriers were placed between mice in order to reduce background and signal bleed over between mice. For all images, mice were placed ventral side facing up in order to maximize signal detection from abdominal organs. For computed tomography (CT) images, mice were placed on the CT carrier and imaged using diffuse light imaging tomography (DLIT) and CT functionality.

After euthanasia, the abdominal wall of the mice was removed and imaging was repeated. To image individual organs, the gallbladder, liver, spleen and caecum were removed and placed in a sterile petri dish. Each organ is then homogenized in sterile PBS and plated for c.f.u. enumeration as described above. c.f.u. were normalized to the mass of tissue and reported as c.f.u. mg^−1^.

### Image quantification

Bioluminescent images were analysed using Living Image 4.7.4 software. After image capture, multiple images were combined into a single file or image sequence, and all further analyses were done with images normalized to the same radiance scale. Luminescence was quantified in units of radiance (p s^−1^ cm^−2^ sr^−1^) in order to compare photons (p) detected between images with different areas (cm^2^) and duration of exposure (s). In order to subtract any background signal, the lower end of the scale was set to the highest amount of signal detected in the negative control mice, as any signal detected in this image reflects baseline autoluminescence. In order to quantify luminescence in specific areas of the mice, regions of interest (ROIs) were drawn around foci of luminescence and data were exported to GraphPad Prism 9 for analysis.

## Results

### Fitness of bioluminescent *S.* Typhimurium

**Fig. 2.**
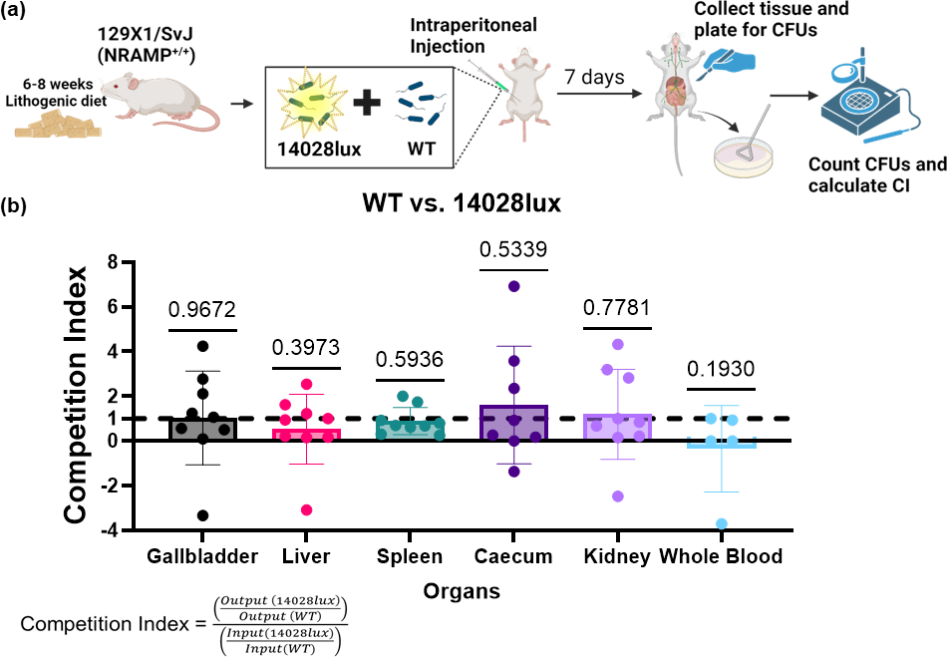
Comparison of fitness between bioluminescent *S*. Typhimurium to WT in murine infection. (**a**) *S*. Typhimurium 14,028 (WT) was transduced with the *lux* operon from the *S*. Typhimurium Xen33 strain from Perkin Elmer©, creating 14,028 lux. 129X1/SvJ mice were fed a lithogenic diet for 6–8 weeks to induce gallstone formation. After cessation of diet, these mice were infected with equal c.f.u. of WT and 14,028 lux isolates via an i.p. injection. Seven days after the initial infection, the mice were sacrificed, and the gallbladder, liver, spleen, kidney, caecum and whole blood were removed. Created with Biorender.com. (**b**) Organs were homogenized, and c.f.u. per milligram of tissue were quantified and compared via a competition index. Data of the gallbladder, liver, spleen, caecum and kidney represent *n*=2 independent experiments. Data from the whole blood reflect data from one experiment. Note: one caecal sample was excluded due to technical errors. Data were analysed via a one-sample t-test to a hypothetical mean of 1.0 and significance level of 0.05. Resulting *P*-values are presented above.

*S*. Typhimurium Xen33 contains a chromosomal insertion of the *Photorhabdus luminescens lux* operon, allowing for stable, constitutive expression of bioluminescence [28]. Xen33 is derived from the FDA 1189 strain of *S*. Typhimurium [28]. Therefore, to allow for direct comparison with *S*. Typhimurium 14,028 infection in mice, *S*. Typhimurium 14,028 was transduced with the *lux* operon of *S*. Typhimurium Xen33 to create ‘14,028 lux’. With whole-genome sequencing, we were able to identify the presence of a >7.8 kb chromosomal insertion in 14,028 lux (Fig. S1, available in the online Supplementary Material). Importantly, the 3′ region of the insert is a kanamycin-resistant gene as predicted given that the Xen33 *lux* construct is preceded by a kanamycin-resistant cassette. The insertion of the *lux* operon is directly upstream of *tomB* but does not interrupt the operon (Fig. S1). TomB is a component of the Hha-TomB toxin-antitoxin system [29,31]. Given this mutation, we wanted to confirm that 14,028 does not have any changes in fitness in our murine model, in comparison to the WT strain. We infected 129X1/SvJ mice with equal amounts of WT and 14,028 lux bacteria and, 7 days after infection, measured the amounts of each strain in the gallbladder, liver, spleen, caecum, kidney and blood of each mouse (Fig. 2a). We then calculated a competition index for each organ and found that there was no significant difference between c.f.u. mg^−1^ of either strain in any organs (Fig. 2b).

### Detection of early infection

**Fig. 3.**
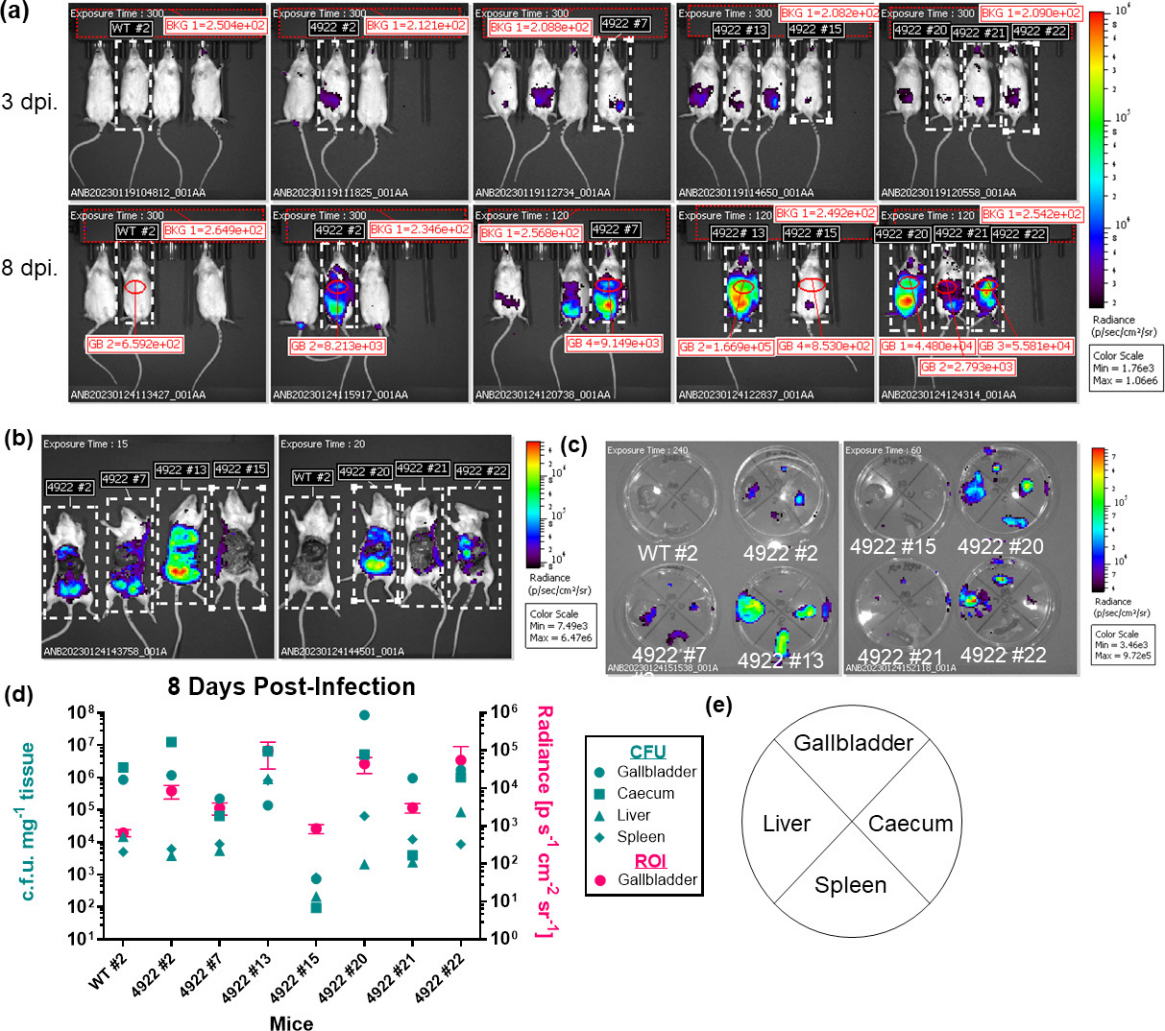
Visualization and validation of bioluminescent *Salmonella* in live mice at 3 and 8 dpi. 129X1/SvJ mice were fed a lithogenic diet for 6–8 weeks to promote chronic infection. Mice were infected via an i.p. injection of 1.1×10^4^–1.6×10^4^ c.f.u of either WT *S*. Typhimurium (WT labelled mice) or *S*. Typhimurium transduced with a *lux* operon (14,028 lux; 4922 labelled mice). (**a**) Mice were anaesthetized via isoflurane inhalation and then imaged with the IVIS Spectrum CT at 3 and 8 dpi. Images in the same column represent the same mice imaged at each time point indicated by the date to the left of the row. Mice were maintained in the same position for each image, and mice selected for further analysis at 8 dpi can be identified by the label and white dashed box surrounding the subject mouse. All images are normalized to the same radiance scale (p s^−1^ cm^−2^ sr^−1^), and the lower limit of the scale has been fixed to the maximum radiance detected on the abdomens of the control WT mice in order to subtract baseline auto-luminescence. The average radiance within an ROI centred around each mouse’s gallbladder (in red) is included in the 8 dpi images. Background (BKG) in each image is included to detect potential highly erroneous signals produced from the stage or manifold that may affect the overall signal. The exposure time (seconds) is included in each image, and the radiance colour scale is included to the right of the images. (**b**) At 8 dpi, the indicated mice were euthanized, and the abdominal wall was removed to directly image internal organs. (**c**) The gallbladder, liver, spleen and caecum were removed and imaged in Petri dishes. The radiance colour scale is included to the right of the images. The orientation of the organs in petri dishes is indicated in (**e**). (**d**) The gallbladder, liver, spleen and caecum from the respective mice were homogenized and plated for c.f.u. per milligram of tissue. The c.f.u. per milligram of tissue are compared with the average and sd radiance of the gallbladder ROI calculated in the respective images in (**a**).

We have shown that mice infected with WT *S*. Typhimurium accumulate bacteria in their gallbladder and other organs within the first week of infection [15,26, 27]. Therefore, we wanted to determine if we can detect these bacteria early in infection with *in vivo* imaging. We injected 1.1×10^4^–1.6×10^4^ c.f.u. of either WT or 14,028 lux in sterile PBS into the peritoneal cavity of mice pre-fed a lithogenic diet. Three and 8 days after infection, we anaesthetized and imaged the mice. Notably, the WT infected mice do not produce any signal over background at either time point, and most of the 14,028 lux mice have a visibly more intense signal at 8 days in comparison to 3 days (i.e. 4922 #2, 7, 13, 20, 21 and 22) (Fig. 3a). At 8 days post-infection (dpi), we quantified the bioluminescent signal from the gallbladders of mice indicated in the ROIs in Fig. 3a. To confirm the source of signal and localization of the gallbladder ROIs in Fig. 3a, we then euthanized the specific mice indicated in Fig. 3a and removed their abdominal wall to directly visualize the intra-abdominal cavity (Fig. 3b). The gallbladder, liver, spleen and caecum were then isolated and imaged in Petri dishes (Fig. 3c and e) prior to being homogenized in sterile PBS in order to enumerate c.f.u. mg^−1^ present in each organ. We were able to detect luminescence from living mice infected with 14,028 lux but not from the non-luminescent WT infected mouse (Fig. 3a). This remained true after directly imaging the intra-abdominal cavity and individual organs, confirming that the observed luminescent signal is intra-abdominal and specific to the 14,028 lux infected mice (Fig. 3b, c, e). Importantly, all the organs of the WT infected mouse had c.f.u. mg^−1^ >10^3^, supporting that the luminescent signal is specific to the 14,028 lux strain, and any luminescent signal from these mice is background or autoluminescent noise (Fig. 3d). The ROIs drawn around the gallbladder region of the mice indicate that some mice had bacteria in their gallbladder, and this is supported by c.f.u. enumeration that shows that all 14,028 lux infected mice with gallbladder c.f.u. mg^−1^>10^3^ also had gallbladder ROI radiances measured at above that of the background (Fig. 3d). These data support that our *in vivo* imaging technique is sensitive enough to detect infection as early as 3 dpi and that it is specific to luminescence from the bacteria.

### Monitoring infection progression

**Fig. 4.**
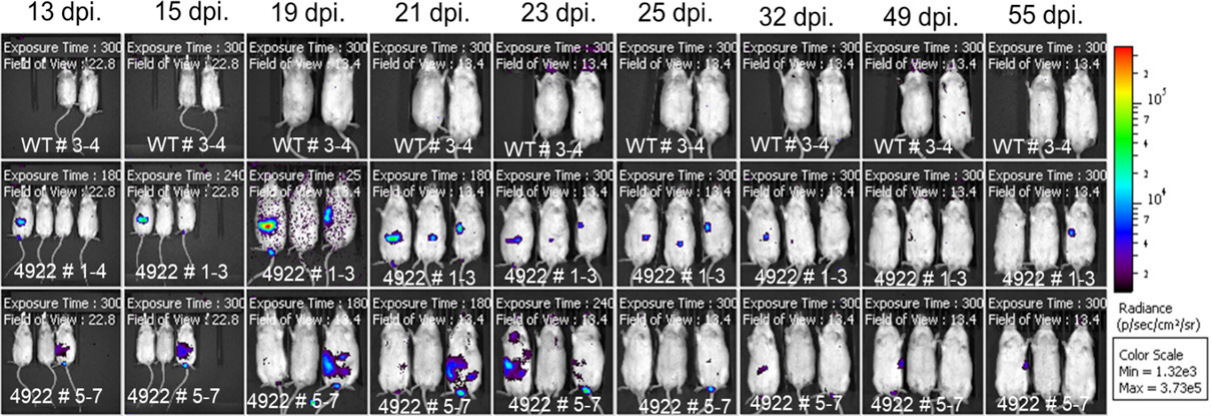
Serial visualization of bioluminescent *Salmonella* in mice from 23 to 55 dpi. 129X1/SvJ mice were fed a lithogenic diet for 6–8 weeks to promote chronic infection. Mice were infected via an i.p. injection of 1.1×10^4^–1.6×10^4^ c.f.u. of either WT *S*. Typhimurium (WT labelled mice) or *S*. Typhimurium transduced with a *lux* operon (14,028 lux; 4922 labelled mice). Mice were anaesthetized via isoflurane inhalation and then imaged with the IVIS Spectrum CT at 13, 15, 19, 21, 23, 25, 32, 49 and 55 dpi. Images in the same row represent the same mice imaged at each time point indicated by the date at the top of the column. Mice were maintained in the same position for each image and can be identified in the order from left to right indicated on the label. All images are normalized to the same radiance scale (p s^−1^ cm^−2^ sr^−1^), and the lower limit of the scale has been fixed to the maximum radiance detected on the abdomens of the control WT mice in order to subtract baseline auto-luminescence. Any condensation on the anaesthesia manifold and nose cones was erroneously detected as a luminescent signal (notably visible in the 23 dpi WT image) and was ignored when normalizing the images. Image exposure time (s) and field of view (cm) are indicated in each individual image. Note: Mouse 4922 #4 was euthanized for reasons unrelated to the study after 13 dpi and so is not present in subsequent images; all other mice remain in the same positions.

Given the confirmation of the sensitivity of our technique, we wanted to determine if we can use *in vivo* imaging to track the progression of infection in our model, specifically the transition to the chronic infection stage. We infected a cohort of lithogenic diet-fed mice with either WT or 14,028 lux and imaged them multiple times over 13–63 dpi (Figs4  5a). This time period reflects the transition from acute (<21 dpi) to chronic infection (>21 dpi) and should also reflect a transition to a primarily gallbladder-focused infection. We observed that the 14,028 lux infected mice show variable levels of luminescence over the time period. Multiple mice (i.e. 4922 #1 and 7) show detectable luminescence in their abdomens earlier in infection that wanes to undetectable levels by 23 dpi (Fig. 4). In contrast, some mice (i.e. 4922 #3 and 5) maintain detectable luminescence throughout the experiment, with periodic reductions below the limit of detection (Fig. 4). This is notable because the transition to chronic gallbladder carriage involves formation of biofilm, a phenotype with low metabolic activity, and occurs in the gallbladder, a low-oxygen environment. These data suggest that we can detect the presumed biofilm state of * S*. Typhimurium in the gallbladder of mice at multiple points throughout the course of infection.

**Fig. 5.**
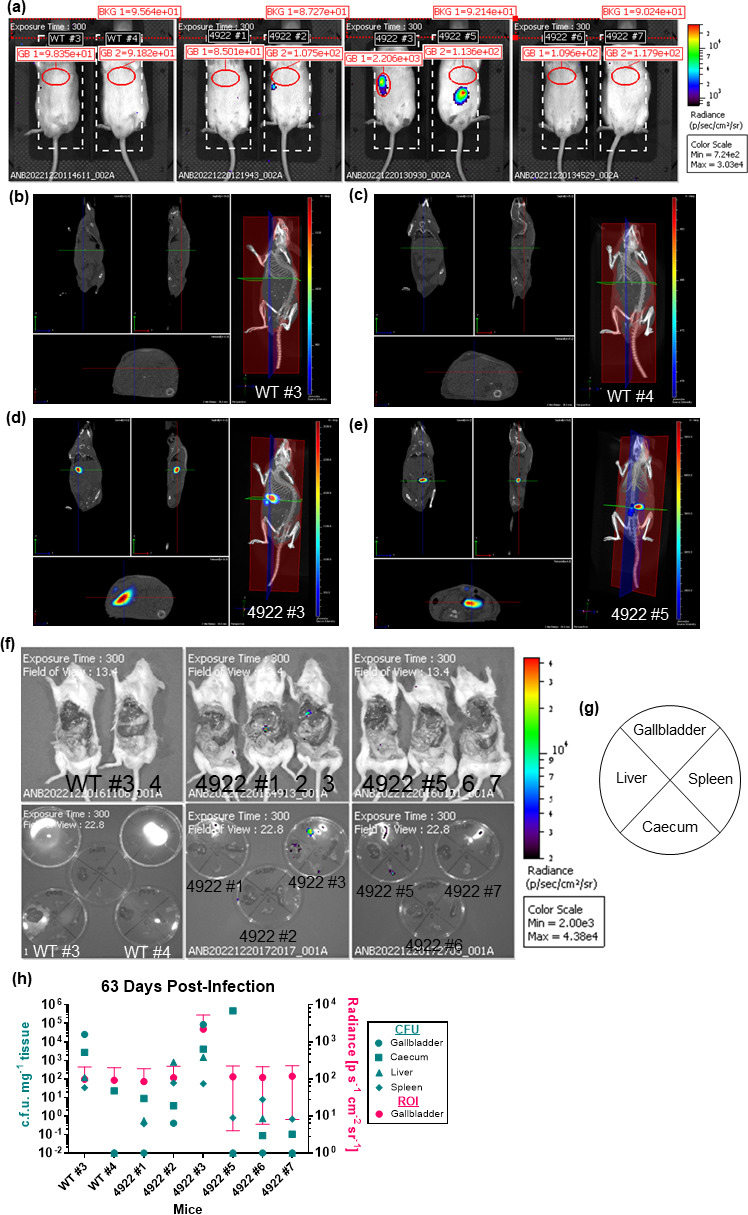
Visualization and validation of bioluminescent *Salmonella* in live mice at 63 dpi. 129X1/SvJ mice were fed a lithogenic diet for 6–8 weeks to promote chronic infection. Mice were infected via an i.p. injection of 1.1×10^4^–1.6×10^4^ c.f.u. of either WT *S*. Typhimurium (WT labelled mice) or *S*. Typhimurium transduced with a *lux* operon (14,028 lux; 4922 labelled mice). (**a**) Mice were anaesthetized via isoflurane inhalation and then imaged with the IVIS Spectrum CT at 63 dpi. The average radiance within a ROI centred around the predicted location of each mouse’s gallbladder (in red) is included in each image. (**b**) WT #2, (**c**) WT #4, (**d**) 4922 #3 and (**e**) 4922 #5 were imaged with CT and DLIT to reconstruct the 3D luminescent source within the subject mouse. The source intensity (p/s) scale is included in each image. Fig. 5 is continued on the following page. (**f**) Mice were then euthanized, and the abdominal wall was removed to directly image internal organs. The gallbladder, liver, spleen and caecum were removed and imaged in Petri dishes. The radiance colour scale is included to the right of the images. The orientation of the organs in petri dishes is indicated in (**g**). (**h**) The gallbladder, liver, spleen and caecum from the respective mice were homogenized and plated for c.f.u. per milligram of tissue. The c.f.u. per milligram of tissue are compared with the average and sd of the gallbladder ROI radiance calculated in the respective images in (**a**).

While these 2D images suggest organ-specific localization of our bacteria, especially at later time points, we wanted to visualize the location of the signal in a 3D rendering of our mice. To do so, we employed CT and DLIT to determine the depth of the source signal in our mice. DLIT measures the amount of light emitted through the surface of the animal over a series of wavelengths in order to reconstruct the distribution of the luminescent source inside the subject tissue and then superimposes this over CT images to create a 3D render of the subject. We used this technology to image the mice at 63 dpi and observed that the signal we identified in 2D images in 4922 #3 to be presumptively gallbladder does in fact originate from a depth corresponding to the gallbladder as observed on the CT image (Fig. 5d). The CT and DLIT analysis also suggests that the luminescent source in 4922 #5 is likely caecal (Fig. 5e). Importantly, the CT and DLIT analysis of WT #3 and 4 does not identify any luminescent source (Fig. 5b and c). To further confirm the CT images, we sacrificed the mice and removed their abdominal wall and repeated 2D images. Again, the source signal localized to the gallbladder of 4922 #3 *in situ* and to the gallbladder after it was removed and imaged (Fig. 5f and g). We enumerated the c.f.u. in the organs of all mice and compared the gallbladder c.f.u. mg^−1^ to the ‘gallbladder’ ROIs drawn on the live mouse images. The presence of >10^5^ c.f.u. mg^−1^ of 14,028 lux correlated with a gallbladder-specific radiance above the background (Fig. 5h), which is higher than the limit of detection observed in Fig. 3d, but may be explained by lower numbers in this case. This confirms that we are able to identify bioluminescent bacteria in the gallbladder using a combination of CT and DLIT imaging. This also confirms that we can differentiate mice that are infected with 14,028 lux from those that have cleared infection.

## Discussion

Operons conferring bioluminescence are naturally present in select species of bacteria, and these have been transferred to pathogenic bacteria of interest in order to induce luminescence [32,35]. Multiple luminescent constructs have been developed to facilitate imaging, but their use requires luciferase enzymes and luciferin substrate, oxygen and ATP [33]. The genes responsible for luciferase production are typically inserted into the bacterial chromosome to prevent selective plasmid expulsion or deleterious overexpression. Luciferin may be administered exogenously to the animals to trigger detectable light production. However, our technique employs a more efficient approach by including all the genes necessary so that the bacteria constitutively produce light. This approach also likely increases the probability of detection of bacteria in biofilms, given that it does not rely on diffusion of the luciferin substrate into the biofilm. Moreover, this approach takes advantage of the heterogeneity of phenotypes within the biofilm to circumvent the limited metabolic activity in the biofilm. Given that the operon is constitutively expressed, any cells that are more metabolically active, and consequently producing more ATP, will produce light, effectively highlighting this subset of the biofilm population for optimal detection.

The introduction of bioluminescent *in vivo* imaging has been an important tool that provides an alternative to the time- and resource-expensive methods of tissue collection to evaluate infection status. Additionally, this technology reduces the amount of mice necessary to complete an experiment, given that the same mouse can be observed over multiple time points rather than having to use multiple cohorts of mice. Multiple groups have used similar approaches to study *Salmonella* infection in murine models [2,18, 36]; however, these efforts have focused on acute infections or food safety. By applying this technique to a chronic typhoidal murine model, we expand our ability to study the pathogenesis of and potential novel therapeutics for chronic typhoidal infection. This study expands on this model by enabling us to noninvasively and accurately detect infection without sacrificing the mice. Using this model, we have determined that luminescence can be detected 3 dpi and that infectious burden can be quantitated with the use of imaging software to measure light radiance in ROIs. Importantly, with this technology, we can observe luminescence localizing to the gallbladder after 21 days and persisting up to 63 dpi, confirming the chronic carriage state in an, otherwise, asymptomatic mouse. The combination of CT and bioluminescent imaging in our model is particularly powerful because it increases confidence in the localization of infection in specific organs, rather than general body cavities.

*S*. Typhi primarily persists in human chronic carriers by forming biofilms on gallstones in the gallbladder. Importantly, in a minority of cases, there may be foci of chronic infection in other locations outside of the gallbladder that, despite treatment, continue the carriage state [37,39]. It is difficult to study the natural timeline of carriage in humans, and it is currently unknown what the maximum time of gallbladder carriage is and the proportion of human patients who may spontaneously clear infection without medical intervention. The use of *in vivo* imaging in our murine model has allowed us to explore this question. We have determined that about one out of six infected mice maintain infection in the gallbladder long term. This is important for understanding the characteristics of this model and suggests that carriage may be similarly dynamic in human patients. However, it has yet to be determined why some mice clear infection, while others remain persistently infected. This information may provide tools for treatment that can be translated to human patients. The use of *in vivo* imaging in this murine model will allow for differentiation of infection status, and future studies can use this technique to explore the immune status and efficacy of novel therapeutics at distinct infection states.

## Abbreviations

CT: computed tomography
DLIT: diffuse light imaging tomography
dpi: days post-infection
GI: gastrointestinal
i.p.: intraperitoneal
Kan: kanamycin
LB: Luria–Bertani
ROI: regions of interest
